# HPV-associated moderately differentiated squamous cell carcinoma of the cervix with pathologically confirmed bladder invasion and radiologically presumed adrenal metastasis in a patient with prior lung adenocarcinoma resection: a rare case report and clinical discussion

**DOI:** 10.3389/fonc.2026.1874217

**Published:** 2026-07-10

**Authors:** Yaxiong Tang, Yanbo Dai, Min Jing, Kang Wang

**Affiliations:** 1 Department of Urology, The First People’s Hospital of Neijiang, Neijiang, China; 2 Department of Pathology, The First People’s Hospital of Neijiang, Neijiang, China; 3 Department of Radiology, The First People’s Hospital of Neijiang, Neijiang, China

**Keywords:** bladder invasion, cervical cancer, HPV, obstructive uropathy, squamous cell carcinoma, urinary diversion

## Abstract

**Background:**

Cervical squamous cell carcinoma (CSCC) rarely presents with urinary system symptoms as the initial manifestation. When the tumor invades the bladder trigone, it may mimic primary urologic disease. We report a case with pathologically confirmed bladder invasion.

**Case:**

A 74-year-old woman with a history of left lung adenocarcinoma resection (pT1bN0M0, stage IA2) presented with anuria, bilateral hydronephrosis, and fever. Initial workup revealed acute kidney injury (creatinine 720.5 μmol/L) and severe urinary tract infection. Emergency bilateral ureteral stenting and transurethral bladder biopsy were performed. Transurethral biopsy showed squamous epithelial hyperplasia with focal atypia; immunohistochemistry (IHC) revealed CD34+, GATA3 focal weak+, CK7+, CK20−, P63+, Ki-67 15%, insufficient for definitive malignancy. Persistent hydronephrosis prompted laparoscopic exploration in July 2023. A biopsy taken from the vesicouterine junction (interface between bladder and uterus) showed invasive squamous cell carcinoma with diffuse p16 positivity (CK7+/CK20−/P63+), providing pathological confirmation of bladder wall invasion by cervical cancer. Gynecologic consultation then identified a 2.5 cm cervical mass with contact bleeding, and cervical biopsy confirmed moderately differentiated CSCC with diffuse p16 block positivity. Staging imaging demonstrated a radiologically presumed isolated 4.2 × 2.5 cm right adrenal metastasis without other distant metastases. Final clinical diagnosis: HPV-associated CSCC, clinical FIGO stage IVB (pathologically confirmed bladder invasion, radiologically presumed adrenal metastasis without histologic confirmation). The patient underwent palliative ileal conduit urinary diversion, which provided short-term renal function improvement; she died 2 months after definitive diagnosis due to widespread disease progression.

**Conclusion:**

CSCC presenting with obstructive uropathy is rare and easily misdiagnosed. Gynecologic history taking and pelvic examination are imperative for female patients, and should be performed promptly, especially in the setting of adjacent organ lesions. The combination of imaging evidence of anatomical continuity and CK7+/CK20−/P63+/p16+ IHC profile has auxiliary value for differential diagnosis. Comprehensive staging including adrenal evaluation is essential. Early palliative urinary diversion may provide temporary relief in selected patients.

## Introduction

Cervical cancer is the fourth most common malignancy in women worldwide, with squamous cell carcinoma accounting for approximately 70% of cases; 99.7% are associated with high-risk HPV (predominantly types 16 and 18) (1). Classic presenting symptoms include postcoital bleeding and abnormal vaginal discharge. Bladder involvement typically occurs in advanced stages (FIGO IIB and above) and is reported in only 5%–8% of CSCC cases (1). However, CSCC presenting initially with urinary symptoms due to bladder trigone invasion is extremely rare and frequently misdiagnosed as primary urologic disease, leading to delayed oncologic treatment (2). For female patients with unexplained bladder trigone lesions, gynecologic etiology should be routinely considered, as urinary symptoms are often secondary to local invasion of primary gynecologic malignancies.

Adrenal metastasis from cervical cancer is uncommon, with an incidence of less than 5% in advanced CSCC (1). Such metastases are often asymptomatic and detected incidentally. Bladder invasion indicates locally advanced disease and complicates local treatment planning (1), and concomitant distant metastasis such as adrenal involvement further adversely affects overall prognosis (3). A prior history of non-gynecologic malignancy, particularly lung cancer, further elevates the diagnostic complexity of differentiating primary bladder squamous neoplasms (4).

Herein, we report a rare case of HPV-associated moderately differentiated CSCC presenting initially with obstructive uropathy due to bladder trigone invasion and bilateral ureteral orifice involvement, complicated by isolated adrenal metastasis at diagnosis, in a patient with a history of resected lung adenocarcinoma. This case highlights diagnostic challenges, the auxiliary value of combined imaging and IHC markers, and the palliative role of urinary diversion.

## Case presentation

A 74-year-old woman presented to the urology department in May 2023 with a 1-month history of progressive urinary difficulty, initially attributed to neurogenic bladder, followed by acute anuria, lower abdominal pain, and fever (38.9 °C) for 3 days. Her medical history included left upper lobe lung adenocarcinoma resection in March 2021 (pT1bN0M0, stage IA2) with no evidence of recurrence on over 2 years of follow-up. She had no prior gynecologic screening, no HPV testing, and no HPV vaccination. Gynecologic history: postmenopausal for 22 years, no abnormal vaginal bleeding or discharge; gravida 3, para 2, all spontaneous vaginal delivery. No smoking or alcohol use; no family history of malignancy.

Physical examination at admission: Temperature 38.9 °C, pulse 112/min, respiratory 22/min, blood pressure 135/78 mmHg. Lower abdomen mildly tender with no rebound tenderness, bladder area dull on percussion. External genital examination unremarkable.

Initial laboratory tests: severe urinary tract infection (WBC 17.52 × 10^9^/L), acute kidney injury (creatinine 720.5 μmol/L, urea 28.6 mmol/L), electrolyte disturbances (hyponatremia 125 mmol/L, hypochloremia 92 mmol/L, hypocalcemia 1.8 mmol/L, hyperphosphatemia 2.3 mmol/L). Procalcitonin 6.94 ng/mL, CRP 128 mg/L. Urinary ultrasound showed bilateral hydronephrosis (right 2.3 cm, left 2.8 cm). Non-contrast pelvic CT (contrast was not administered due to severe AKI) showed diffuse bladder wall thickening and a soft tissue lesion in the trigone, but the cervix was not clearly visualized. The chest CT showed no evidence of lung cancer recurrence. Adrenal CT was not performed initially, as the clinical priority was focused on managing the acute urinary tract obstruction and urosepsis.

**Figure 1:**
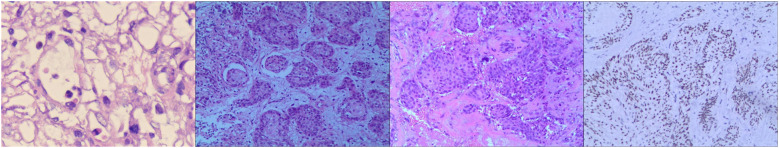
Initial transurethral bladder biopsy (May 2023). From left to right: H&E showing squamous epithelial nests with focal atypia (×100); P63 IHC (nuclear positivity, ×100); CK7 IHC (cytoplasmic positivity, ×100); Ki-67 IHC (≈15%, ×100). Additional IHC confirmed CD34+, GATA3 focal weak+, CK20−. p16 was not performed on this specimen.

Emergency bilateral ureteral double-J stenting and transurethral resection of bladder tumor (TURBT) with biopsy were performed on May 20, 2023. Intraoperatively, diffuse tumor-like mucosal lesions were noted in the trigone and ureteral crest. Transurethral bladder biopsy showed focal squamous epithelial nests with mild to moderate atypia; IHC: CD34+ (vascular), GATA3 focal weak+, CK7+, CK20−, P63+, Ki-67 15% (**Figure 1**). No invasive carcinoma was identified, and p16 IHC was not performed on this specimen.

Post-operative abdominal CT incidentally revealed a 3.0 × 1.9 cm right adrenal mass, initially interpreted as possible adenoma. The patient’s infection and renal function improved with antibiotics; she was discharged with creatinine 210 μmol/L.

**Figure 2:**
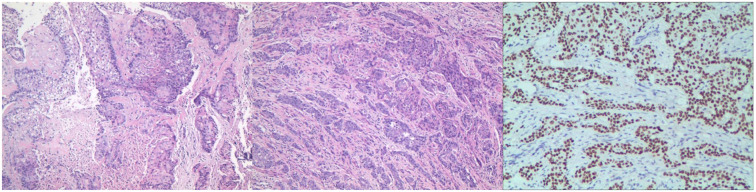
Biopsy of the vesicouterine junction obtained during laparoscopic exploration (July 2023). From left to right: H&E showing invasive squamous cell carcinoma (×200); P63 IHC (nuclear positivity, ×200); CK7 IHC (cytoplasmic positivity, ×200). Additional stains confirmed CK20− and diffuse p16 block positivity (not shown). This specimen provides pathological confirmation of bladder wall invasion by cervical cancer.

In July 2023, the patient was readmitted for persistent hydronephrosis and recurrent anuria. Follow-up abdominal CT (performed after renal function partially recovered, with creatinine 210 μmol/L) revealed heterogeneous thickening of the bladder wall with moderate to marked enhancement on contrast-enhanced phases, which was highly suggestive of a bladder neoplasm. The uterus was reduced in size with scant cervical calcification, and no definite abnormalities were identified in the bilateral adnexal regions. The right adrenal mass had enlarged to 4.2 × 2.5 cm during the interval. On July 15, emergency ureteral stent exchange was performed. On July 25, after multidisciplinary team (MDT) consultation, laparoscopic pelvic exploration and ileal conduit urinary diversion were performed due to extensive pelvic adhesions, recurrent life-threatening urinary obstruction, and concern for underlying pelvic malignancy. The choice of ileal conduit over percutaneous nephrostomy was based on patient/family preference to avoid chronic external catheters for quality-of-life reasons. During laparoscopy, a biopsy was taken from the vesicouterine junction (the interface between the posterior bladder wall and the anterior uterine wall). Histopathology revealed invasive squamous cell carcinoma with squamous differentiation. IHC showed CK7+, CK20−, P63+, and diffuse p16 positivity (**Figure 2**). In comparison, the transurethral bladder mucosal biopsy obtained in May 2023 only revealed severe squamous dysplasia, with no definitive evidence of invasive carcinoma identified. This vesicouterine junction biopsy provided pathological confirmation of bladder wall invasion by cervical cancer. Postoperative creatinine improved to 172.2 μmol/L.

**Figure 3:**
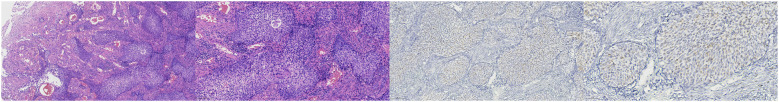
Cervical biopsy (September 2023). From left to right: H&E showing moderately differentiated SCC (×100); H&E higher magnification (×200); p16 IHC – diffuse block positivity (×200); Ki-67 IHC (≈30%, ×200).

Although cervical origin was first suspected in July 2023 based on the IHC profile of the vesicouterine junction biopsy (CK7+/CK20−/P63+/p16+), the patient initially refused gynecologic examination. She only agreed to evaluation in September 2023. Gynecologic consultation was then initiated. Gynecologic examination: speculum showed atrophic cervix with an irregular 2.5 cm cauliflower-like mass on the posterior lip, friable with contact bleeding; uterine body atrophic with poor mobility; bilateral parametria were thickened but did not extend to the pelvic sidewall. Cervical biopsy confirmed moderately differentiated invasive squamous cell carcinoma with diffuse p16 block positivity (**Figure 3**), consistent with high-risk HPV-associated carcinogenesis (5). Ki-67 was 30% in the cervical lesion (vs. 15% in the transurethral bladder biopsy).

In October 2023, abdominal CT showed that the right adrenal mass measured approximately 6.4 × 4.8 cm. No biopsy was performed due to the patient’s poor general condition and concomitant coagulation dysfunction (prothrombin time [PT] prolonged by 3.2 seconds, international normalized ratio [INR] 1.4). Plain and contrast-enhanced pelvic MRI revealed an irregular mass-like abnormal signal lesion in the cervical region, with a maximum three-dimensional dimension of approximately 5.1 × 4.6 × 9.6 cm. The lesion involved the uterine body, vagina, and adjacent sigmoid colon, accompanied by bilateral iliac lymphadenopathy, which was highly suspicious for cervical cancer. Chest CT demonstrated no evidence of lung cancer recurrence.

Final diagnosis: HPV-associated moderately differentiated squamous cell carcinoma of the cervix with pathologically confirmed bladder invasion (vesicouterine junction biopsy, p16+) and radiologically presumed isolated right adrenal metastasis. According to FIGO 2021, pathologically confirmed bladder invasion qualifies as stage IVA; the adrenal lesion, if confirmed, would be IVB. Given the lack of histopathological confirmation of the adrenal lesion, the final clinical FIGO stage is designated as IVB, based on pathologically confirmed bladder invasion and radiologically suspected adrenal distant metastasis. The patient had an ECOG performance status of 3 and declined systemic chemotherapy/immunotherapy after full discussion of goals of care (symptom relief and quality of life). The patient had already undergone palliative ileal conduit urinary diversion in July 2023 to relieve obstruction and preserve renal function. After definitive diagnosis, the patient and her family declined systemic anti-tumor therapy including chemotherapy, targeted therapy, or immunotherapy, and opted for best supportive care only.

The patient’s disease progressed rapidly after definitive diagnosis. Follow-up CT on October 20, 2023 showed extensive disease progression: large pelvic mass, adrenal mass with IVC invasion, new pelvic ascites, and left sacral bone lesion suspicious for metastasis. On November 5, 2023, she developed acute respiratory failure and cardiopulmonary arrest. Resuscitation was initiated but the family declined intubation and further aggressive measures. The patient died shortly thereafter due to widespread metastatic disease and respiratory failure.

A detailed timeline of clinical events is provided in **Supplementary Table 1**.

## Discussion

This case presents several unique and clinically significant features: (1) CSCC presenting initially with obstructive uropathy due to bladder trigone invasion and ureteral orifice involvement, with bladder invasion pathologically confirmed by vesicouterine junction biopsy, and the cervical primary verified by cervical biopsy; (2) a radiologically presumed isolated adrenal metastasis; (3) prior history of lung adenocarcinoma complicating differential diagnosis; and (4) the auxiliary value of combined imaging and IHC markers.

### Clinical uniqueness and diagnostic challenges

CSCC typically presents with gynecologic symptoms. Bladder involvement occurs in only 5%–8% of advanced cases (1), and presentation with urinary symptoms as the initial manifestation is exceedingly rare. In this case, the patient initially presented with anuria and bilateral hydronephrosis to the urology department, and there was a 4-month delay before the definitive diagnosis of cervical squamous cell carcinoma was confirmed. This diagnostic delay resulted from multiple objective factors: (1) Atypical presentation – no vaginal bleeding; initial non-contrast CT failed to visualize the cervical mass. (2) Acute life-threatening conditions – urosepsis and AKI prioritized obstruction relief and infection control. (3) Sampling limitation – transurethral bladder biopsies showed only dysplasia without invasive carcinoma, limiting early tumor etiology investigation. (4) Patient refusal of gynecologic examination – although cervical origin was suspected in July 2023 based on the IHC profile of the vesicouterine junction biopsy (CK7+/CK20−/P63+/p16+), the patient initially refused gynecologic evaluation. She only agreed to and underwent gynecologic examination and cervical biopsy in September 2023, which definitively identified the cervical primary.

Importantly, a biopsy of the vesicouterine junction during laparoscopic exploration revealed invasive SCC with diffuse p16 positivity, providing pathological confirmation of bladder invasion. This finding indicates that when transurethral bladder mucosal biopsy fails to yield a definitive diagnosis, more thorough evaluation of perivesical tissues and adjacent pelvic organs is warranted. For female patients in particular, dedicated gynecologic workup is essential to clarify the underlying etiology.

The isolated adrenal lesion further increased diagnostic complexity. Adrenal metastases from cervical cancer are rare and often detected incidentally during staging (3). In this patient, the adrenal mass was initially missed due to focus on acute urinary symptoms; later identified on follow-up CT and considered metastatic based on interval growth, heterogeneous enhancement, and absence of other obvious primary lesions. Additionally, the previously resected lung adenocarcinoma was of glandular origin, histologically inconsistent with the squamous histology of the confirmed pelvic primary tumor; combined with the absence of pulmonary recurrence on chest CT, lung cancer-derived adrenal metastasis was further ruled out. However, no biopsy was performed, so the diagnosis remains radiologically presumed.

### Immunohistochemistry in tumor origin determination

Distinguishing cervical SCC with bladder invasion from primary bladder SCC is important. The combined immunophenotype CK7+/CK20−/P63+ supports squamous epithelial origin but does not independently identify the cervix as primary. P16 IHC was not performed on transurethral bladder mucosal biopsies. However, the vesicouterine junction biopsy showed diffuse p16 positivity, consistent with HPV-associated carcinogenesis (5). The cervical lesion also showed diffuse p16 block positivity. Primary bladder pure SCC typically shows variable CK7/CK20, is consistently P63+, and is usually p16-negative (non-HPV-associated). Primary bladder urothelial carcinoma with squamous differentiation typically shows diffuse GATA3 positivity, which was only focally weak in our case (6, 7). The definitive identification of the cervix as the primary site was based on comprehensive clinicoradiologic-pathologic correlation: Anatomical continuity on MRI – cervical mass directly extending to bladder trigone. Cervical biopsy – moderately differentiated SCC with diffuse p16 positivity. Vesicouterine junction biopsy – invasive SCC with p16 positivity, confirming direct bladder invasion from the cervical primary. The difference in Ki-67 proliferation index (30% in cervical lesion vs. 15% in transurethral biopsy) reflects tumor heterogeneity and does not indicate different primaries. Thus, the CK7/CK20/P63/p16 panel has auxiliary diagnostic value but must be interpreted together with imaging and site-specific pathology.

### Practical diagnostic workflow for unexplained obstructive uropathy in women

Based on this case and existing evidence, we propose the following practical diagnostic workflow (derived from clinical experience, not a validated guideline):

1. Initial evaluation: Comprehensive history (including gynecologic), physical examination, renal function, and cross-sectional imaging (pelvic MRI if contrast contraindicated).
2. Cystoscopy with biopsy of visible bladder lesions; simultaneous vaginal speculum examination and cervical inspection recommended.
3. IHC panel for bladder squamous lesions: CK7, CK20, P63, GATA3, p16, Ki-67.
4. If bladder lesion shows squamous histology with CK7+/CK20−/P63+, prompt gynecologic consultation and cervical biopsy, even without gynecologic symptoms.
5. If transurethral biopsies are non-diagnostic but clinical suspicion high, consider biopsy of the vesicouterine junction during laparoscopy or image-guided deep biopsy.
6. Comprehensive staging imaging including adrenal evaluation.

### Management considerations

For advanced CSCC with bladder invasion and distant metastasis, curative resection is rarely feasible. For advanced pelvic malignancies with urinary tract obstruction, the core treatment goals are symptom palliation, renal function preservation, and quality of life maintenance (4, 8, 9). The actual benefit of urinary diversion is influenced by multiple factors, including tumor burden, expected survival, baseline patient condition, and treatment goals (4, 9). In this patient, palliative ileal conduit diversion provided temporary relief of obstruction and short-term improvement in renal function, but overall survival benefit was limited due to rapid tumor progression. The patient had poor performance status (ECOG 3) and coagulopathy; after goals-of-care discussion, she and her family declined systemic therapy and opted for best supportive care. MDT collaboration (urology, gynecologic oncology, radiology, pathology, palliative care) is essential.

### Limitations

This report has limitations inherent to a single-center, retrospective case study. First, HPV genotyping was not performed, precluding identification of the specific HPV genotype. Second, pathological confirmation of the adrenal metastasis was not obtained due to the patient’s poor clinical condition, and the diagnosis was based on imaging features and clinical course. Third, transurethral superficial bladder mucosal biopsies only revealed squamous dysplasia without definitive invasive carcinoma due to sampling limitation; bladder wall invasion was confirmed by deep biopsy at the vesicouterine junction obtained during laparoscopic exploration. Finally, long-term follow-up was not available due to the patient’s rapid disease progression and short survival after diagnosis.

## Conclusion

This case highlights an extremely rare presentation of HPV-associated cervical squamous cell carcinoma with initial obstructive uropathy due to bladder trigone invasion and bilateral ureteral orifice involvement, complicated by isolated radiologically presumed adrenal metastasis at diagnosis. Key clinical lessons: CSCC should be considered in women with unexplained obstructive uropathy and bladder squamous lesions. The CK7+/CK20−/P63+/p16+ IHC panel has auxiliary diagnostic value, but final diagnosis relies on clinicoradiologic-pathologic correlation. Comprehensive staging imaging, including adrenal evaluation, is essential. Early palliative urinary diversion may provide temporary relief and renal function improvement in selected patients. Increased awareness of this atypical presentation and systematic diagnostic evaluation may reduce delays and improve outcomes.

## Data Availability

The original contributions presented in the study are included in the article/**Supplementary Material**. Further inquiries can be directed to the corresponding author.

## Supplementary material

Supplementary Table 1 Timeline of clinical events, interventions, and key outcomes (provided as separate file).
