# Supplementary material for: HPV-associated moderately differentiated squamous cell carcinoma of the cervix with pathologically confirmed bladder invasion and radiologically presumed adrenal metastasis in a patient with prior lung adenocarcinoma resection: a rare case report and clinical discussion
Source: Front Oncol. 2026 Jul 10;16:1874217. doi: 10.3389/fonc.2026.1874217 (PMC13395741; doi:10.3389/fonc.2026.1874217)
Supplement: Supplementary Table 1 — Timeline of clinical events, interventions, and key outcomes (provided as separate file). [file Table1.doc]

**Supplementary Table 1.** Timeline of clinical events, interventions, and key outcomes.

| **Date** | **Clinical Events** | **Interventions** | **Key Findings/Outcomes** |
| --- | --- | --- | --- |
| March 2021 | History of left upper lobe lung adenocarcinoma resection | Surgical resection of left upper lobe lung adenocarcinoma | Pathological diagnosis: pT1bN0M0, stage IA2; no recurrence on 2-year follow-up |
| May 2023 (1 month prior to admission) | Progressive urinary difficulty, initially attributed to neurogenic bladder | None (symptomatic management) | Symptoms gradually worsened over 1 month |
| May 17, 2023 | Acute anuria, lower abdominal pain, fever (38.9°C); emergency admission | Laboratory tests, urinary system ultrasound, pelvic CT, chest CT | Severe urinary tract infection (white blood cell count 17.52 × 10⁹/L), acute kidney injury (creatinine 720.5 μmol/L, urea 28.6 mmol/L), electrolyte disturbances (hyponatremia 125 mmol/L, hypochloremia 92 mmol/L, hypocalcemia 1.8 mmol/L, hyperphosphatemia 2.3 mmol/L), elevated procalcitonin (6.94 ng/mL) and C-reactive protein (128 mg/L); bilateral hydronephrosis (right 2.3 cm, left 2.8 cm); bladder trigone soft tissue mass; no lung recurrence |
| May 20, 2023 | Emergency urologic intervention due to persistent anuria and hydronephrosis | Bilateral ureteral double-J stenting, transurethral resection of bladder tumor (TURBT) with biopsy | **Bladder trigone soft tissue lesion. Pathology: squamous epithelial hyperplasia with focal atypia (IHC: CK7+/CK20−/P63+, Ki-67: 15%, p16 not performed); insufficient for definitive malignancy** |
| Post-May 20, 2023 | Postoperative recovery and follow-up | Antibiotics for infection control, supportive care for renal function improvement | Infection resolved; renal function improved (creatinine 210 μmol/L); abdominal CT incidentally identified right adrenal mass (3.0 × 1.9 cm, initially interpreted as adrenal adenoma); patient discharged |
| July 2023 | Readmission due to persistent hydronephrosis and recurrent anuria | Repeat abdominal/pelvic CT, emergency ureteral stent exchange | Right adrenal mass enlarged to 4.2 × 2.5 cm; hydronephrosis persisted despite prior stenting |
| July 25, 2023 | **Palliative intervention for recurrent obstruction and diagnostic laparoscopy** | Laparoscopic pelvic exploration, ileal conduit urinary diversion, **biopsy of vesicouterine junction** | Extensive pelvic adhesions. **Biopsy of vesicouterine junction showed invasive squamous cell carcinoma with diffuse p16 positivity (CK7+/CK20−/P63+), providing pathological confirmation of bladder wall invasion by cervical cancer**; postoperative creatinine improved to 172.2 μmol/L. **Cervical origin suspected, but patient initially refused gynecologic examination.** |
| September 2023 | **Gynecologic evaluation for unexplained squamous lesion (after patient consented)** | Cervical examination, cervical biopsy, pelvic MRI, adrenal CT | **Patient agreed to evaluation after prior refusal. Gynecologic exam: 2**.5 cm irregular cervical mass with contact bleeding; cervical biopsy: **moderately differentiated CSCC (p16 block positive, Ki-67: 30%)**.  **MRI confirmed cervical mass with direct bladder trigone invasion. Adrenal CT confirmed isolated right adrenal mass (radiologically presumed metastasis).** |
| October 20, 2023 | Routine follow-up for disease monitoring | Chest/abdominal/pelvic CT | Extensive disease progression: large pelvic mass with indistinct uterine and adnexal structures; right adrenal mass with adjacent inferior vena cava invasion; new findings: pelvic ascites, left sacral bone hyperdense lesion (suspicious for bone metastasis); persistent bilateral hydronephrosis |
| November 5, 2023 | Acute respiratory failure, cardiopulmonary arrest | Resuscitation (declined by family) | Patient died due to widespread metastatic disease and respiratory failure |
